# Barriers to and Facilitators of the Use of Digital Tools in Primary Care to Deliver Physical Activity Advice: Semistructured Interviews and Thematic Analysis

**DOI:** 10.2196/35070

**Published:** 2022-08-30

**Authors:** Paulina Bondaronek, Samuel J Dicken, Seth Singh Jennings, Verity Mallion, Chryssa Stefanidou

**Affiliations:** 1 Office for Health Improvement and Disparities Department of Health and Social Care London United Kingdom; 2 Research, Translation & Innovation Public Health England London United Kingdom; 3 Institute of Health Informatics University College London London United Kingdom; 4 Centre for Obesity Research University College London London United Kingdom

**Keywords:** physical activity, capability, opportunity, and motivation—behavior, COM-B, Theoretical Domains Framework, TDF, primary care professionals, health care professionals, physical activity advice

## Abstract

**Background:**

Physical inactivity is a leading risk factor for many health conditions, including cardiovascular disease, diabetes, and cancer; therefore, increasing physical activity (PA) is a public health priority. Health care professionals (HCPs) in primary care are pivotal in addressing physical inactivity; however, few HCPs provide PA advice to patients. There can be obstacles to delivering PA advice, including lack of time, confidence, or knowledge. Digital technology has the potential to overcome obstacles and facilitate delivering PA advice. However, it is unknown if and how digital tools are used to deliver PA advice in primary care consultations and what factors influence their use.

**Objective:**

We aimed to understand the use of digital tools to support primary care consultations and to identify the barriers to and facilitators of using these systems.

**Methods:**

Overall, 25 semistructured interviews were conducted with primary care HCPs. Professionals were sampled based on profession (general practitioners, practice nurses, and health care assistants), prevalence of long-term conditions within their practice area, and rural-urban classification. The data were analyzed thematically to identify the influences on the use of digital tools. Themes were categorized using the COM-B (capability, opportunity, and motivation—behavior) model and the Theoretical Domains Framework to identify the barriers to and facilitators of using digital tools to support the delivery of PA advice in primary care consultations.

**Results:**

The identified themes fell within 8 domains of the Theoretical Domains Framework. The most prominent influence (barrier or facilitator) within psychological capability was *having the skills to use digital tools*. *Training in the use of digital tools* was also mentioned several times. The most notable influences within physical opportunity were *limited digital tools to prompt/support the provision of PA advice*, *time constraints*, *efficiency of digital tools, simplicity and ease of use* of digital tools, and *integration with existing systems*. Other physical opportunity influences included *lack of access to digital tools* and *technical support in the use of digital tools*. Within social opportunity, a notable barrier was that *digital tools reduce interpersonal communication with patients*. *Patient preference* was also identified. Several important influences were within reflective motivation, including *confidence to use digital tools, beliefs about the usefulness of digital tools*, the *belief that digital tools “are the way forward*,” *beliefs related to data privacy and security concerns*, and *perceptions about patient capabilities*. About automatic motivation, influences included *familiarity and availability* regarding digital tools and the fact that digital tools *prompt behavior*.

**Conclusions:**

A variety of influences were identified on the use of digital tools to support primary care consultations. These findings provide a foundation for designing a digital tool addressing barriers and leverages the facilitators to support PA advice provision within primary care to elicit patient behavior change and increase PA.

## Introduction

### Background

Physical inactivity is a leading cause of death and noncommunicable disease worldwide [1]. Being physically active can reduce the risk of all-cause mortality and help prevent and manage a wide range of long-term conditions, including cardiovascular disease (CVD), some cancers, and neurodegenerative diseases [2,3]. Moving from a state of inactivity to meeting the UK government physical activity (PA) recommendations of 150 minutes per week of moderate to vigorous PA can reduce the risk of CVD incidence by 17%, CVD mortality by 23%, and type 2 diabetes incidence by 26%, even after adjusting for body weight [4]. Furthermore, PA has positive impacts on mental health and well-being [2,3].

Therefore, identifying effective methods to increase PA in the population is of great importance. One approach is to provide PA advice to patients in primary care. As a trusted source of health-related information that frequently interacts with large proportions of the population, health care professionals (HCPs) within primary care have pivotal roles in encouraging greater PA [5,6]. As many as 1 in 4 people say they would be more active if they were advised by a general practitioner (GP) or a nurse.

Delivering brief PA advice in primary care has been shown to be cost-effective [7,8], with positive impacts on PA and health outcomes [9,10]. As such, the National Institute for Health and Care Excellence recommends that brief PA advice be provided in primary care [11]. However, delivery of brief PA advice in primary care is not routine and remains to be fully established. Only one-third of all patients report receiving such advice [5,12]. Despite knowledge among HCPs that increasing PA is beneficial for their patients [13], a number of reviews and studies have identified key obstacles that limit the delivery of PA advice in primary care. Important obstacles include a lack of knowledge (of national PA guidelines, of how to deliver advice, of what advice to give, and of how to communicate effectively) [6,9,13-15], a lack of tools or resources [6], an inability to follow-up on patients [6,13], the perceived readiness and motivation of the patient to change [6,16], and lack of confidence and time constraints [6,13,17].

Establishing the routine delivery of PA advice in primary care requires overcoming such obstacles. A promising avenue is the use of digital tools, which may provide opportunities to facilitate the delivery of PA advice in primary care. These can include electronic tools that are integrated within clinical information technology systems in primary care or stand-alone technology that can help facilitate and signpost patients to various resources. The World Health Organization has highlighted the importance of using innovative digital technologies to promote PA and reduce sedentary behavior in its Global Action Plan on Physical Activity [18].

Digital tools have previously been used to deliver PA advice in primary care [19-21] either by supporting [22] or replacing [23] face-to-face delivery of PA advice. Digital tools appear to have potential utility in increasing PA by supporting the delivery of PA advice [19,24,25]. However, primary care HCPs have mixed views on the usability of digital tools, with barriers to their use including technical issues and complexity, disruption to service workflow, and increasing workload [19,26-28].

### Objective

To determine the value of digital tools to support the delivery of PA advice and how to optimize their development and integration, it is important to fully understand the existing challenges of delivering PA advice, the influence on using digital tools, the required characteristics of digital tools, and the opportunities to incorporate digital tools into existing practice. However, there is a paucity of evidence surrounding the obstacles facing the use of digital tools to deliver PA advice. Studies rarely use a behavioral framework to systematically identify barriers and facilitators or instead focus on patient perspectives [19]. Studies have considered only specific digital tools, such as eHealth or mobile health (mHealth) interventions, and not all potential digital tools.

Systematic approaches to investigating the factors that influence health-related behaviors and professional practices can be facilitated using behavioral science tools. The capability, opportunity, and motivation—behavior (COM-B) system is a model of behavior change that helps to understand the influences on performance of a behavior [29]. A related model is the Theoretical Domains Framework (TDF), which can be mapped onto COM-B to further categorize influences into the facilitators that increase, and the barriers that hinder, the behavior [30]. COM-B and TDF have been widely used in previous studies to synthesize findings on barriers and facilitators for a range of behaviors, including a review of physician-reported barriers to using evidence-based recommendations for low back pain [31], a review of the factors influencing the implementation of screening and brief interventions for alcohol in primary care [32], and specifically for promoting PA by HCPs [16]. In this study, we used COM-B and TDF to systematically map the barriers to and facilitators of using digital tools to deliver PA advice in primary care.

We aimed to understand the use of digital tools to support primary care consultations and to identify the barriers to and facilitators of using these systems to deliver PA advice.

The specific objectives were (1) to gain insights into the use of digital tools within primary care settings to understand the influences on their use to deliver PA advice and (2) to systematically map the influences of using COM-B and TDF to understand the barriers to and facilitators of using digital tools within primary care to deliver PA advice.

## Methods

### Study Design

This was an exploratory qualitative study drawing on interviews with HCPs in primary care.

### Sample

A sample of HCPs was recruited purposively (by a third-party recruiter) based on profession (GPs, practice nurses, and health care assistants [HCAs]), prevalence of long-term conditions within the area (in particular, obesity, depression, hypertension, coronary heart disease, and diabetes), and rural-urban classification. During recruitment, HCPs were also screened to ensure a range of experience levels (based on the length of time working in primary care, self-reported levels of delivering PA advice, and self-reported digital skills). Data collection ceased once saturation of themes was reached, resulting in a total of 25 interviews being completed, transcribed, and coded.

### Inclusion Criteria

To be included, study participants had to be a GP, nurse, or HCA; must have worked in general practice; must have worked in the United Kingdom health care system for a minimum of 1 year; must be an English speaker; and must be aged 18≥ years.

### Data Collection

Semistructured interviews lasting 60 to 90 minutes were conducted via telephone in March and April 2020. This time frame coincided with the introduction of the first COVID-19 pandemic protocols in the United Kingdom, including the national lockdown on March 23, 2020. Hence, all study interviews were conducted via telephone. In line with ethical guidelines, written informed consent was obtained from the participants before commencing the interview. A topic guide based on COM-B [33] was used by the interviewers to support discussions.

During interviews, HCPs were asked a series of open-ended questions about their routines and working days; the systems and resources they use routinely to identify patients and to deliver and record advice; their capability, opportunity, and motivation to use these systems and resources effectively; any barriers to using these tools and resources; and suggested solutions and improvements to overcome them.

The topic guide included various prompts and follow-up questions to help elicit data relevant to the research question.

### Data Management and Analysis

The interviews with the 25 HCPs were recorded on password-protected and encrypted machines to ensure data privacy and security. The recordings were uploaded to the encrypted, password protected Citrix platform to be transcribed verbatim by a third-party provider, and the original recordings were then deleted from the study team’s systems. The third-party provider removed any identifying information during the transcription and returned anonymized transcripts to the study team for data analysis.

The anonymized transcripts were imported into Microsoft Excel for analysis. Participant responses were broken down into constituent parts to analyze distinct thoughts and ideas independently. Content analysis, informed by the COM-B model, was used to analyze the data. One researcher (VM) read each of the 25 transcripts, extracted data relevant to the use of digital tools, and inputted the data into an Excel spreadsheet. In this study, we defined a “digital tool” as any use of information and communications technology to support HCPs in primary care to deliver PA advice. This definition was adopted from the World Health Organization’s definition of digital health [34].

In total, 165 comments from the participants relating to the use of digital tools were recorded. Another member of the research team inductively coded the data line by line using constant comparison techniques within and between codes to ensure that they accurately reflected the material. Codes were then examined for similarities and grouped inductively into themes regarding barriers to and facilitators of using digital tools to identify patients and to deliver and record PA advice. The themes that emerged from this process, that is, the ones that were identified as being important, were either articulated by multiple respondents (high frequency) or were articulated particularly clearly and forcefully (elaboration) or both. Once the data were coded as a barrier, facilitator, or both; they were deductively classified under the COM-B model [29] and TDF [35] to systematically understand these behaviors and needs. When multiple COM-B components and themes could be used to code data, further data segmentation was considered if it was deemed that the existing data segment contained discrete thoughts. Further data segmentation was reported during coding by putting a forward slash (/) between the COM-B components and the themes.

Classifying data into COM-B components followed expert guidelines [33]. One researcher (AG) was tasked with classifying all the extracts, and a second researcher (SSJ) coded 20% of the extracts to highlight and resolve any discrepancies in the coding. A random number generator was used to provide a random sequence of Excel cell numbers containing data segments that would be coded by SSJ. After independently completing one round of coding, AG and SSJ met via video calls to discuss codes. Any disagreements over codes were discussed until consensus was reached, and the data set was updated accordingly. Similarly, the decision to split the data segments was discussed between researchers until an agreement was reached.

### Ethics Approval

Ethical approval for this study was provided by the Public Health England Research Ethics and Governance Group (#NR0181). Participants provided written informed consent before taking part.

## Results

### Participant Summary

Participant characteristics are presented in Table 1. Participants tended to be practice nurses or HCAs, older, and working in an urban setting, with a range of primary care experience.

The barriers to and facilitators of using digital tools to deliver PA advice in primary care are presented in Table 2. Additional participant responses are presented in Multimedia Appendix 1. Important themes were identified within psychological capability, physical and social opportunities, and reflective and automatic motivation.

**Table 1:** Summary characteristics of participants recruited for interviews (n=25; 23 respondents for age and 24 respondents for location).

Characteristic		Participants
**Profession, n (%)**		
	General practitioner	6 (24)
	Practice nurse	10 (40)
	Health care assistant	9 (36)
**Age (years; n=23, 92%), n (%)**		
	18-30	3 (13)
	31-50	7 (30)
	50+	13 (57)
**Primary care experience, n (%)**		
	1-10 years	7 (28)
	11-20 years	9 (36)
	20+ years	9 (36)
**Location (n=24, 96%), n (%)**		
	Rural	7 (29)
	Suburban	1 (4)
	Urban	16 (67)

**Table 2:** Important themes identified by participants during interviews on the barriers to and facilitators of using digital tools to deliver physical activity advice in primary care.

COM-B^a^ and Theoretical Domains Framework			Themes
**Capability**			
	**Psychological**		
		Knowledge skills	Having the skills to use digital tools Training in the use of digital tools
	**Physical capability**		
		Physical skills	Not reported as an influence
**Opportunity**			
	**Physical**		
		Environmental context and resources	Availability Efficiency of digital tools Integration with existing systems Lack of access to digital tools Limited digital tools to prompt or support the provision of physical activity advice Simplicity and ease of use Technical support in the use of digital tools Time constraints
	**Social**		
		Social influences	Digital tools reduce interpersonal communication Patient preferences
**Motivation**			
	**Reflective**		
		Beliefs about capabilities	Confidence to use digital tools Perceptions about patient capabilities
		Beliefs about consequences	Beliefs about the usefulness of digital tools Beliefs related to data privacy and security Belief that digital tools are “the way forward”
	**Automatic**		
		Reinforcement emotions	Familiarity Prompt behavior

^a^COM-B: capability, opportunity, and motivation—behavior [29,35].

### Psychological Capability

*Having the skills to use digital tools* was reported by numerous respondents as an important factor influencing the use of digital tools in primary care to deliver PA advice, being described as both a barrier and a facilitator. Although some respondents reported feeling confident in their digital skills and ability to use digital tools, a number of HCPs discussed how not having the skills and/or confidence to use digital tools may act as a barrier to their use. Although providing appropriate training may facilitate the use of digital tools within primary care, HCPs overwhelmingly discussed the lack of adequate training to provide them with the skills and confidence to use digital tools (barriers). Hence, *training in the use of digital tools* was identified as another notable theme. Because of the lack of formal training, some staff members discussed having to rely on other members of staff within the practice with more experience using digital tools to teach them how to use the systems. Therefore, HCPs may benefit from some form of training on how to use digital tools:

I’m pretty good but EMIS is one of those things that there is always something more to learn really. You can learn the basics in quite a short period of time but I am still finding things that I think, God, if I’d have known that a few years ago, that would have saved me an awful lot of time.

Nurse, 31-50 years

There is no formal training by and large, other than you may get sent a document of how to do something. So we have relied upon one of our staff members who, for want of a better word, is like an IT manager who will take overall charge of these things and oversee their introduction and development, and disseminate that information as a practice and ensure that we’re all up to speed. So you need to have one person who has that as their responsibility and role within the practice.

General practitioner, ≥50 years

### Physical Opportunity

Physical opportunity was the most frequently coded COM-B component (Table 1). A substantial barrier to the use of digital tools in the provision of PA advice in primary care was the lack of digital options to prompt or support the delivery of PA advice; this was coded under the theme *limited digital tools to prompt/support the provision of PA advice*. Some interviewees cited existing templates that initiate discussion of PA, whereas several others said that they were not aware of *any* digital tools that prompt or support the provision of PA advice. Specifically, diabetes-related templates and National Health Service Health Check templates prompt PA questions or advice (and may lead patients to a program that increases their PA):

I think it’d be quite useful really because with EMIS you can see people’s BMI and results and things like that, so it would be quite useful to have a prompt in the corner to say “encourage physical activity” or blah blah blah. It’s something that would be nice to have.”

Nurse, ≥50 years

It’s not on the diabetic template to ask about physical activity but it is on the NHS [Health Check] to check their physical activity.

Nurse, ≥50 years

We use templates [in our consultations]... If there is a patient with pre-diabetes, we... ask them: “would you like to go for the diabetes prevention programme?”... There’s no other template for us to use... On the system for the diabetes... we need to tell them... [to do] exercise—either walking or... going to the gym or any sort of exercise at home.

Nurse, ≥50 years

Another important barrier is *time constraints*. It was clear that HCPs often experienced time pressures and did not always have time to consider other ways of working (ie, they will often default to what they are used to). On the flip side, there was a feeling that digital tools could save time by increasing efficiency; hence, the theme of *efficiency of digital tools*. Digital tools have been reported to make more efficient use of the limited time available for consultations, allowing data to be more easily captured and stored in comparison with manual data recording (facilitators):

When you’re so busy and flat out, you don’t have sometimes that time to just sit back and reflect and think, well, is there another way I could be doing this more efficiently?

General practitioner, ≥50 years

You have your clinics. You have your QOFs to do. You want to follow the NICE guidelines on every patient with a long-term condition. We have all of those responsibilities as well as the urgent on the day requests. Jiggling time is always a factor.

Nurse, ≥50 years

Relating to the themes of *efficiency of digital tools* and *time constraints,* was the theme of *simplicity and ease of use*, which may act as either a barrier or facilitator, depending on the design and subsequent functionality of the digital tool. For example, templates need to be as simple as possible, quick to use, and easy to navigate and must also facilitate the collection of all mandated or important data. Another way in which digital tools can be made easier to use is if they allow for *integration with existing systems*. This was also highlighted by the respondents as both a barrier and facilitator. This was categorized as a separate theme, as it was deemed to be a specific requirement. Another physical opportunity barrier to the use of digital tools is the *lack of access to digital tools* in some areas. Another physical opportunity consideration was the presence of *technical support in the use of digital tools*. Respondents reported that having technical support to hand facilitated the use of digital tools:

It’s much easier. Much easier than sitting there writing things out. You can click. It gives you more time to do other things. It gives you more time with the patient. You’re not spending lots of time writing things out. You are more for the patient than you are writing things down.

HCA, 31-50

I think sometimes in general practice the issue is we don’t have much time... So I think any way in which we can reduce the number of clicks, to put it simply, the better, and if this system was generated automatically, it flags it up, then that would be better than having to deal with all those issues and then think about doing something else on top as well. I think the easier to use, the quicker to use, the less steps involved the better really.

General practitioner, 31-50 years

It would have to be something that would be compatible with the system that we’re using, and unfortunately I’m trying to get an ECG machine to be compatible with EMIS. So it’s all about compatibility and whether one talks to the other.

HCA, ≥50 years

The final physical opportunity factor was *availability*, which has been mentioned several times. One respondent, for example, said that they used specific digital tools because it was what was available to them and was what they had always used:

I suppose it’s what we’ve always used, we’ve never been told there’s anything else that can be used.

Nurse, 18-30 years

### Social Opportunity

Barriers and facilitators related to social opportunities were less frequently discussed by the HCPs. However, there was an indication that some HCPs felt that *digital tools reduce interpersonal communications* with patients (a barrier). One respondent also stated that their propensity to use digital tools may be influenced by *patient preferences* (barriers or facilitators):

Part of me doesn’t mind but other times I think, Oh gosh I feel I’m looking at a computer screen rather than looking at a patient. I wasn’t trained to do that; I’m very old school as well because I trained back in the eighties so I don’t mind using it, I appreciate we have to move on with the times, but I don’t like it too much because I find that I’m watching the screen and making sure I’ve got everything that I need to fill on there without actually looking at the patient and just talking to them properly.

Nurse, ≥50 years

### Reflective Motivation

One of the clearest themes under reflective motivation to emerge from the data was that of *confidence to use digital tools*. All respondents who mentioned confidence discussed it as a facilitator, expressing that they had the confidence required to use such systems. However, it is implicit in the notion of confidence that one could just as easily have it as not have it; a lack of confidence would, of course, be a barrier to the use of digital tools. It should be highlighted that *confidence to use digital tools* often goes hand in hand with *having the digital skills to use digital tools*, as having the skills to do something tends to breed confidence, while not being confident may indicate a skill deficit. Despite this overlap, skills and confidence are different influences and are coded differently within COM-B, which explains why some quotes in Multimedia Appendix 1 could seemingly fit into either:

I mean I’m of the generation which is fairly IT savvy, so I feel quite confident.

General practitioner, 31-50 years

Another theme that could be both a barrier and a facilitator for the use of digital tools was *beliefs about the usefulness of digital tools*. If someone believes that a system has utility, they will be more inclined to use it (facilitator), whereas if they believe the system is not useful or is indeed a hindrance, they may be disinclined to use it (barrier). Most respondents who mentioned the usefulness of digital tools within their practice felt that such systems *were* useful, but this was not unanimous. A common belief discussed by HCPs with positive views toward the usefulness of digital tools is that these systems save time and increase efficiency by, among other things, facilitating the sharing of data with secondary health care providers. It was also mentioned that digital tools present unique opportunities to provide varied care to patients digitally when there is limited opportunity to provide physical care to patients face to face. However, others said that templates do not provide useful options and may be too time consuming to use during consultations and that patients might also benefit from having a physical copy of advice:

We can even do things like video consultations now which I think we’ve had to embrace because of the current situation with COVID. I think it will change the way we practise ongoing because we can see the efficiencies of these. I think the model of general practice personally is going to change hugely after this because we can see we can do things safely and differently and more efficiently.

General practitioner, ≥50 years

I don’t find the template is particularly useful... I don’t think it’s very useful in the information that it’s asking for. Then the options it gives you, do you want to refer them to the health trainer? Nearly everybody will say no to that because it’s too involved. It’s too time consuming.

HCA, ≥50 years

A related theme to *beliefs about the usefulness of digital tools* was the *belief that digital tools “are the way forward.”* Sometimes, this theme overlapped with *beliefs about the usefulness of digital tools*; respondents said that digital tools were the way forward and then went on to support this with reasons based on usefulness, but sometimes it appeared to be offered as a reason in its own right. The *belief that digital tools* “*are the way forward*” was a facilitator of the use of digital tools:

The opportunities are there aren’t they, we’re moving forward and everything’s IT and it’s the way forward, for patients as well, apps and doing everything online and using phones.

Nurse, ≥50 years

Beliefs related to data privacy and security was another theme under reflective motivation that emerged from the data. This theme was both a barrier to and a facilitator of the use of digital tools, depending on the particular beliefs of each respondent. Some felt that digital tools improved security around patient data by reducing mistakes, whereas others said that the safety features required to ensure patient safety within digital tools could act as a barrier to their use:

They’re just safer, and they protect patient confidentiality, and they’re safer to use, things we can audit, trails, process it all, and obviously check if anything goes wrong, if there was a fax it may reject or get sent somewhere else if the number was wrong.

HCA, 18-30 years

The final theme under reflective motivation was HCPs’ *perceptions about patient capabilities*, and again, this was both a barrier and facilitator. Several respondents suggested that their inclination to use digital tools during a consultation would depend on the technical capabilities of the patient in question:

My dad, he needs everything explained manually and wouldn’t go near a computer; for him, I’d need to spend more time with him, to discuss a questionnaire I’d need to print it out and go through it with him, even phones.

Nurse, ≥50 years

### Automatic Motivation

A common facilitator of the use of digital tools in primary care within automatic motivation was that digital tools, specifically templates, *prompt behavior*. For instance, templates were described as useful as they provided guidelines and tick boxes to prompt HCPs to ask relevant questions and ensure that nothing was missed during consultations. Another theme under automatic motivation, which was mentioned by a few respondents, was *familiarity*. For example, one respondent highlighted that newly introduced digital tools can be a bit daunting, but that this feeling subsides over time as they become familiar:

They’re optional, yes... I choose to use them, yes... It’s easier and I feel like it’s more thorough, and when it’s a busy day especially, it’s nice to just have that as a prompt.

HCA, 18-30 years

I mean, sometimes when you first learn them, it is a bit daunting. You think, “oh,” and you’re looking through them, but once you’ve done it a few times, you get a rhythm... As I said, if you go through every box, you can’t go wrong.

HCA, 31-50 years

## Discussion

### Principal Findings

This study aimed to investigate the use of digital tools to deliver PA advice. However, we found that digital tools for delivering PA advice were limited. Some templates include PA prompts, but no template focuses specifically on facilitating PA advice. Hence, we considered the use of digital tools in primary care; with the identification of themes based on high frequency, elaboration, or both. This study has implications for the development of digital interventions to facilitate the delivery of PA advice in primary care.

Barriers and facilitators to using digital tools to deliver PA advice identified in this study included *skills and training to use digital tools*; *efficiency of digital tools*, including their *integration with existing systems* and *simplicity and ease of use*; *patient preferences*; *confidence to use digital tools*; *beliefs about the usefulness of digital tools*; *perceptions about patient capabilities*; and *beliefs relating to data privacy and security*. *Limited digital tools to prompt/support the provision of PA advice*, *time constraints*, and the fact that *digital tools* *reduce* *interpersonal communication* were barriers; and the use of digital tools to *prompt behavior*, the *belief that digital tools are* “*the way forward*,” and having *technical support in the use of digital tools* were facilitators.

This qualitative study expands on previous findings on the barriers and facilitators to delivering PA advice in primary care [6,16], using a behavioral framework to systematically identify the barriers to and facilitators of using digital tools to deliver PA advice in primary care. The findings from this study indicate that important influences related to knowledge, time, and confidence in delivering PA advice are also important for using digital tools. However, other themes, including the efficiency and integration of digital tools and data privacy and security concerns are important influences, specifically for using digital tools in this context. As for delivering PA advice, there was variability across HCPs as to whether themes were barriers, facilitators, or both to using digital tools.

The mixed views regarding the usability and utility of digital tools emerging from this study build on previous findings for eHealth interventions to deliver PA advice. Similarly, some HCPs find eHealth interventions useful and easy to use, but others perceive eHealth interventions to be time consuming or ineffective, with technical issues, inexperienced staff, and the complexity of programs as barriers to their use [6,19,20,28].

As with delivering PA advice [6,36], time was a barrier to using digital tools, which is closely related to their efficiency and ease of use. Cumbersome digital tools that are poorly integrated slow down work and disrupt workflow, creating a barrier to their use to support the delivery of PA advice [26,37,38]. A digital tool needs to be simple, easy, and time efficient to fit within short consultations [37,38] but also versatile, given that time constraints are likely to vary depending on consultation length, which may differ between countries. In agreement with the results of this study, integrating an eHealth tool into existing medical programs and workflows is important to facilitate its use [37-39]. The ability of a digital tool to facilitate (or hinder) the delivery of PA advice depends on how simple, effective, and well integrated the system is.

Many participants agreed that digitization was the way forward, providing an efficient, simple, and easy-to-use solution. However, interviews were conducted during the COVID-19 pandemic, when face-to-face consultations were canceled. Before the COVID-19 pandemic, the use of digital tools instead of face-to-face PA advice was previously identified as a barrier in terms of interpersonal communication, with HCPs preferring face-to-face communication [26]. In this study, some participants highlighted that video consultations facilitated giving PA advice to those who were unable to attend in person. In contrast, participants indicated that in-person digital tools could be a barrier if the system excessively detracted from interacting with the patient. Digital tools can facilitate PA advice when they improve communication, which could be achieved by providing templates with recommendations and set phrases or prompts. Digital tools can be used to generate personalized recommendations in an appropriate language to facilitate the delivery of PA advice [37,40], which can be time efficient by using tools before the consultation [37,38,41-43]. mHealth tools can perform important tasks, such as diagnosis, helping to reduce the workload [20]. For example, digital tablets in the waiting room can save time by automating the collection of routine data and performing health screening [39], which is flexible enough to accommodate discussions across varying durations of consultations [19] and support discussions with patients with limited health literacy [44].

### Commentary on the Findings

Previous studies have shown that knowledge, training, or access to educational resources are common barriers and facilitators to delivering PA advice [6,9,45]. This study builds on these findings by indicating that knowledge and training are also barriers and facilitators to using the digital tool itself. The participants pointed out a current lack of technical support for the use of digital tools. There can be educational barriers and technical difficulties in using digital tools such as tablets or apps [38]. Delivering PA advice increases the workload of HCPs [16], and digital tools have the potential to facilitate the delivery of PA advice through improvements in efficiency and ease of use. However, the benefit of a digital tool in reducing workload is likely dependent on the quality of training provided to use digital tools and the resources within it. Digital eLearning systems are already used to provide PA education [46] and training to increase knowledge, confidence, and skills to deliver PA advice, such as the Moving Healthcare Professionals Programme [21]. A systematic review indicated that mobile tools may facilitate PA promotion by addressing knowledge and resource barriers [16] and providing a centralized, integrated tool for easy access to PA resources [47]. However, these systems do not provide training for digital tools. Provision of education and training in digital tools should also be considered if they are being used to support the delivery of PA advice.

Previous studies have identified patient-related factors as an important theme affecting the motivation of HCPs to give PA advice, with patient abilities to use digital tools, preferences, and readiness to change as barriers and facilitators [37]. In this study, patient motivation to change was not an important theme in using digital tools, but HCPs’ perceptions of patient motivation to use a digital tool were a barrier to or facilitator of their use, depending on whether patients preferred physical prints or had the technical capability to use a digital tool. The lack of print materials has also been cited as a barrier to delivering brief PA interventions [36,48]. Importantly, HCPs use their subjective perception of patient capability and motivation to change to determine whether to deliver PA advice [6]. Discussions could be facilitated using digital tools before consultation to assess patient readiness and suitability using a standardized approach [16].

In this study, computer-based interventions were previously proposed to facilitate the delivery of advice by acting as a prompt [39]. The lack of a consistent contextual cue has been a barrier to discussing PA in different contexts [49], whereby structured protocols or templates facilitate the delivery of brief PA advice [36]. Digital tools need to be well integrated, fit in with, and aid the current workflow to be effective prompts [26,40,49]. These are also important themes in this study. PA advice also needs to be delivered in the right context to increase patient receptivity [6]. The ability to adjust the template to suit the consultation and provide a contextual prompt also facilitated the use of a digital tool in this study. Therefore, the use of a digital tool as a prompt requires physical opportunity barriers to be addressed.

In this study, the ability to track and share patient data was considered to be a facilitator to using digital tools. Indeed, the inability to monitor follow-up is a barrier to delivering brief PA advice [6], which could be overcome by using a digital tool to provide a platform to track and monitor patient PA over time at follow-up [37].

One theme in this study, largely unmentioned previously, was that digital tools may prevent mistakes and ensure patient safety by addressing the information gaps in HCPs. In addition, the participants highlighted that the time efficiency of a digital tool may depend on the extent of the safety measures used to ensure patient confidentiality, which may differ across health care systems in different countries.

### Implications for the Development of Digital Tools to Facilitate the Delivery of PA Advice in Primary Care

The results of this study provide several recommendations for the design of a digital tool to support the delivery of PA advice by addressing barriers and leveraging facilitators. First, there appears to be a lack of digital tools that facilitate the delivery of PA advice. We argue that there is an opportunity to develop a digital tool to prompt and guide HCPs to discuss PA with patients. Second, the digital tool should be integrated into the existing workflow of primary care HCPs to reduce any friction and, most importantly, not to produce additional workload for HCPs. Therefore, we recommend developing a relevant contextual prompt at critical points within the consultation to discuss PA. Third, digital tools should facilitate conversations between HCPs and patients. It should be universally applicable to different patients, yet it should give HCPs the freedom to tailor the conversation to the patient. Fourth, the ease, simplicity, and efficiency of digital tools can address some barriers to the delivery of PA advice. However, this requires barriers to using the digital tool itself to also be addressed, such as sufficient education and training in digital tools, confidence in using digital tools, or access to in-house support for using the digital tool. For example, digital tools can be used to generate personalized, printable guides from computer-based assessments of readiness to change and PA levels, as has been recently implemented in the Portuguese National Health Service [42]. The provision of digital templates and eLearning within existing platforms could facilitate HCPs lacking in communication skills or knowledge of PA, with a monitoring system to provide follow-up. Finally, further work should include interdisciplinary collaborations to ensure that the digital tool is usable and efficient and that HCPs engage with it to support the delivery of PA advice in primary care. Hence, the next step should bring together developers who design digital tools in primary care, service users (HCPs) of digital tools to consider the user journey and needs, and behavioral scientists to translate design recommendations into tangible prototypes to be tested.

### Strengths and Limitations

A strength of this study is the use of a behavioral framework for interviewing and analysis to systematically identify the barriers to and facilitators of using digital tools to deliver brief PA advice. The study also asked participants to consider any digital tool where many previous studies have focused on certain aspects of digital tools, such as eHealth or mHealth interventions.

The limitations include the range of HCP specialisms in this study, which included GPs, nurses, and HCAs and therefore did not consider the views of other HCPs within primary care. Furthermore, this study was conducted during the initial months of the COVID-19 pandemic, which may have influenced perceptions of using digital tools. Finally, barriers and facilitators to using digital tools to support the delivery of PA advice in primary care may differ across health care systems in different countries. Hence, the results from this UK study might not be applicable to other national health care systems. However, digital health care tools are becoming increasingly common worldwide, and similar issues have been identified across health care systems.

### Conclusions

Using a behavioral framework and qualitative approach, this study systematically identified important barriers and facilitators to using digital tools to support the delivery of PA advice in primary care. Important themes were found within 8 theoretical domains, most often within physical opportunity. These barriers can be addressed by designing efficient and flexible digital support tools to facilitate HCPs in delivering PA advice in primary care. To do so, future work should combine designers, service users, and behavioral scientists to design and develop testable prototypes.

## Appendix

Multimedia Appendix 1 Summary of the barriers and facilitators on the use of digital systems to deliver physical activity advice in primary care.

## Abbreviations

COM-B: capability, opportunity, and motivation—behavior
CVD: cardiovascular disease
GP: general practitioner
HCA: health care assistant
HCP: health care professional
mHealth: mobile health
PA: physical activity
TDF: Theoretical Domains Framework
