# Supplementary material for: Barriers to and Facilitators of the Use of Digital Tools in Primary Care to Deliver Physical Activity Advice: Semistructured Interviews and Thematic Analysis
Source: JMIR Hum Factors. 2022 Aug 30;9(3):e35070. doi: 10.2196/35070 (PMC9472053; doi:10.2196/35070)
Supplement: Multimedia Appendix 1 [file humanfactors_v9i3e35070_app1.docx]

# **Multimedia Appendix 1**

Table 1: Summary of the barriers and facilitators on the use of digital systems to deliver PA advice in primary care.

| **COM-B** | **TDF** | **Themes** | **Representative quote** |
| --- | --- | --- | --- |
| Psychological Capability | Knowledge  Skills | Having the skills to use digital systems | *“I’m pretty good but EMIS is one of those things that there is always something more to learn really. You can learn the basics in quite a short period of time but I am still finding things that I think, God, if I’d have known that a few years ago, that would have saved me an awful lot of time.”* **(Nurse, 31-50 years)**  *“I think there’s lots of things I don’t know how to use, but maybe 6 or 7 out of 10 systems that I know I need to know and know it well, but I don’t know how to do complicated searches and format templates or that kind of thing, hidden amazing things you can do.”* **(GP, 31-50 years)**  *“I’m happy using them for what I do. I’m not very technical I must admit, I didn’t go into nursing to use computers, but I would say I’m quite ok with what I need to know..”* **(HCA, 50+ years)**  *“I would say I’m very confident. I’ve been using it two years now. So I am quite confident with it. I would say it’s taken me two years to learn how to use it.”* **(HCA, 18-30 years)**  *“I would like to use it more... I mean, I have apps on my own phone and things like that, but apps in work… I haven’t got as many. Maybe I should have more or maybe I don’t know about them. It’s probably the not knowing - not realising that they are there because no one has told me.”* **(HCA, 50+ years)** |
|  |  | Training in the use of digital systems | *“Again, with all these things, if you get good training on it then that’s really, really useful. I think if you throw something at somebody and say, ‘Here you are. Get on with that.’, it can take them longer to work it all out than if you did just say, ‘Right, here’s a fifteen minute video.’ Or whatever it might be. ‘This is how you access the tool’ or whatever it is. ‘This is where we’re going to put it. This is how you access it.’ I think there are a wide variety of technical skills out there in people.”* **(Nurse, 31-50 years)**  *“I feel like sometimes things are just thrown in at us and it’s kind of just expected for us to get on with it. You know, even when accuRX started it was kind of like, ‘oh just deal with it’ kind of thing. I mean I don’t find it that difficult and, again, I use the fake patient to play with. But I don’t feel like there’s much of an explanation or teaching to staff on how to use things.”* **(Nurse, 18-30 years)**  *“There is no formal training by and large, other than you may get sent a document of how to do something. So we have relied upon one of our staff members who, for want of a better word, is like an IT manager who will take overall charge of these things and oversee their introduction and development, and disseminate that information as a practice and ensure that we’re all up to speed. So you need to have one person who has that as their responsibility and role within the practice.”* **(GP, 50+ years)**  *“They gave us a really good introduction and we got training on how to use it, so that was good.”* **(HCA, 18-30 years)** |
| Physical Capability | Physical skills | Not reported as a significant influence |  |
| Physical Opportunity | Environmental context and resources | Efficiency of digital systems | ***“****I think that digital is quick and it’s easy.... I think it is the way forward. I really do. I think the more digital savvy we get, because it’s time consuming, paper saving, time saving. So yes, I think it’s definitely the way forward..”* **(Nurse, 31-50 years)**  *“So I use, for example, something called RightBreathe, which mainly I use for my asthmatic patients. So in terms of – there’s YouTube clips on how they use their inhalers, because I don’t have all the inhalers in-house to demonstrate how to use it. So I will show them a clip on the website, a YouTube clip, and I can also text it to them with accuRX so that they have it on their phone. If they forget how to take it then it’s on their phone.”* **(Nurse, 18-30 years)**  *“It’s much easier. Much easier than sitting there writing things out. You can click. It gives you more time to do other things. It gives you more time with the patient. You’re not spending lots of time writing things out. You are more for the patient than you are writing things down.”* **(HCA, 31-50)**  *“There’s sometimes a battle with IT, sometimes I can’t get the template up because of IT issues and it’s a wasted clinic - a barrier because when it goes down you’ve lost it.”* **(Nurse, 50+ years)**  *“I think it’s the way forward, it’s the way people work, we’re getting people to take pictures of their skin lesions and we can view them, download them and put it in their notes, so you’ve got evidence of what we saw, and we’ve got skype consultations, especially doing the covid19 work and it might change the way we do general practice, and whether we really need to see all those people.”* **(GP, 50+ years)** |
|  |  | Integration with existing systems | *“I suppose because everyone uses the same system, so you know, the data can get just put onto the patient notes on that template and everyone can see it, so you don’t need to be on a certain computer to have a certain software to see…”* **(Nurse, 31-50 years)**  *“There’s certain things preloaded into our system, so whether it was for measuring parameters for risk of CVD, you can link to resources like patient.co.uk, so we use those but not external apps or anything like that.”* **(GP, 31-50 years)**  *“... it would have to be something that would be compatible with the system that we’re using, and unfortunately I’m trying to get an ECG machine to be compatible with EMIS. So it’s all about compatibility and whether one talks to the other.”* **(HCA, 50+ years)** |
|  |  | Lack of access to digital systems | *“I think personally very good, but because this is a deprived area, we struggle with digitisation of all these things.”* **(GP, 50+ years)**  *“Not needing to visit, that’s really handy, the only problem is the people who haven’t got access.”* **(GP, 50+ years)** |
|  |  | Simplicity and ease of use | *“So I like it to flow. I like the template to work. I like it to have an order that works for me in how I would run a consultation. So yes, it might have something at the start, and then it might have height, weight, blood pressure. I like to do things in an order so I actually re-jiggled my template to run in an order that prompts me and makes sure I cover everything that seems a bit logical.”* **(Nurse, 31-50 years)**  *“I think there are only so many things on a screen that you can put. You have to keep things- I suppose you can’t overload with a big screen that you’re looking at with all the details, because I think too much information can actually make you blind to the screen. So you just want a few little reminders.*” **(GP, 50+ years)**  *“I think whatever tool we have would have to be simple. A) for me to use and b) for me to be able to show the patient in an easy way so they understand what we’re showing them. So I think simplicity always wins with me, because it’s easier to understand something if you’re trying to get - if you know what you’re trying to get across to them, the patient will understand me.”* **(HCA, 50+ years)**  *“I think sometimes in general practice the issue is we don’t have much time… So I think any way in which we can reduce the number of clicks, to put it simply, the better, and if this system was generated automatically, it flags it up, then that would be better than having to deal with all those issues and then think about doing something else on top as well. I think the easier to use, the quicker to use, the less steps involved the better really.”* **(GP, 31-50 years)** |
|  |  | Technical support in the use of digital systems | *“We used to be Vision… in the last 3 years we’ve been using SystmOne. It's OK, it's good, we are getting there. If something happens, they come and help us.”* **(Nurse, 50+ years)**  *“Usually if the practice manager has a problem putting it in, she gets our IT girl to do it which is-, she puts everything where it needs to go, so there are not usually any problems.”* **(HCA, 31-50 years)** |
|  |  | Time constraints | *“When you’re so busy and flat out, you don’t have sometimes that time to just sit back and reflect and think, well, is there another way I could be doing this more efficiently?”* **(GP, 50+ years)**  *“So EMIS templates are fairly- they are a bit time consuming but they are fairly easy to build.”* **(Nurse, 31-50 years)**  *“You have your clinics. You have your QOFs to do. You want to follow the NICE guidelines on every patient with a long-term condition. We have all of those responsibilities as well as the urgent on the day requests. Jiggling time is always a factor.”* **(Nurse, 50+ years)**  *“I mean we’ve only got 20 minutes so we are limited to what tools we can use.”* **(Nurse, 50+ years)** |
| Social Opportunity | Social Influences | Digital systems reduce interpersonal communication | *“Part of me doesn’t mind but other times I think, Oh gosh I feel I’m looking at a computer screen rather than looking at a patient. I wasn’t trained to do that; I’m very old school as well because I trained back in the eighties so I don’t mind using it, I appreciate we have to move on with the times but I don’t like it too much because I find that I’m watching the screen and making sure I’ve got everything that I need to fill on there without actually looking at the patient and just talking to them properly.*” **(Nurse, 50+ years)** |
|  |  | Patient preferences | *“So it’s just whatever the patient’s choice is really. Some patients want to know and they want to read up and they’re interested. Other patients don’t. If someone is newly diabetic, we offer them the choice, do you want to go to the diabetic education course? That’s up to them and then we can refer them then.”* **(HCA, 50+ years)** |
| Reflective Motivation | Beliefs about capabilities | Beliefs related to data privacy and security concerns | *“Systems go down, we have power cuts, you have to make sure things are encrypted, that you’re not breaking data laws, etc, and are you talking to the right person, how do you identify them, you’ve got a mobile number but how do you know if they’re answering things electronically? Like any telephone conversation.”* **(GP, 50+ years)**  *“They’re just safer, and they protect patient confidentiality, and they’re safer to use, things we can audit trails, process it all, and obviously check if anything goes wrong, if there was a fax it may reject or get sent somewhere else if the number was wrong.”* **(HCA, 18-30 years)**  *“I think the main barriers really are just consent from patients and safety for their data.”* **(GP, 31-50 years)** |
|  |  | Confidence to use digital systems | *“I mean I’m of the generation which is fairly IT savvy, so I feel quite confident.”* **(GP, 31-50 years)**  *“I’d like to think I’m confident in it.”* **(HCA, 50+ years)**  *“Yes. Very confident. I’ve been using them for a long while now. I’m very confident with using the templates on EMIS.”* **(HCA, 50+ years)** |
|  |  | Perceptions about patient capabilities | *“My dad, he needs everything explained manually and wouldn’t go near a computer for him, I’d need to spend more time with him, to discuss a questionnaire I’d need to print it out and go through it with him, even phones.”* **(Nurse, 50+ years)**  *“I think it’s because it has to be a mutual thing, the patient has to be more compliant, more knowledgeable about it, and I feel that is lacking.”* **(GP, 50+ years)** |
|  | Beliefs about consequences | Beliefs about the usefulness of digital systems | *“I don’t find the template is particularly useful.... I don’t think it’s very useful in the information that it’s asking for. Then the options it gives you, do you want to refer them to the health trainer? Nearly everybody will say no to that because it’s too involved. It’s too time consuming.”* **(HCA, 50+ years)**  *“I think that emphasis of paternalism which used to be the case with medicine and general practising in particular, I think it is out the window now. I think patients want to feel more involved in their care and we’re very much taught, when we’re doing our GP training, to try and give people options and then patients are allowed to make what we would consider the wrong choice. So I think having things like their own references to go away and look at or their own diagrams or pictorial evidence, or video evidence, I think it all adds to the picture of whether or not a patient will likely continue with that health change or not.”* **(GP, 31-50 years)**  *“I think why we use it is because it’s easier and rapid and it’s supposed to be more secure, and we’re in shared care now, alongside the country as well, so people have access to shared care and we work in a multidisciplinary team where electronic records come straight onto the your screen, it saves a lot of time, and data collection, several reasons really. Audit trails and it’s a better service to use electronically, for medication and for general really.”* **(Nurse, 50+ years)**  *“I think you save time, more patients are seen, more queries are answered, in this climate we’re short of GP time, and it adds to safety.”* [In what ways does it add to safety?] “*Well looking at things, to be accurate, with the video consultation it’s nice to see a rash and get a decision.”* **(GP, 50+ years)**  *“We can even do things like video consultations now which I think we’ve had to embrace because of the current situation with COVID. I think it will change the way we practise ongoing because we can see the efficiencies of these. I think the model of general practice personally is going to change hugely after this because we can see we can do things safely and differently and more efficiently.”* **(GP, 50+ years)** |
|  |  | Belief that digital systems are ‘the way forward’ | *“The opportunities are there aren’t they, we’re moving forward and everything’s IT and it’s the way forward, for patients as well, apps and doing everything online and using phones,”* **(Nurse, 50+ years)**  *“If you’d have asked me four weeks ago, I would have said, yes, I’m a bit set in my ways and I like to use telephones and face to face. I think one thing COVID may have done is actually revolutionise the way, I guess not just medicine, but how we operate as a society, and becoming, everyone has suddenly had to become more IT literate and IT savvy, which I think will change things.*” **(GP, 50+ years)**  *“I think it’s something that we need to embrace really, something we have to be careful around, especially around sharing data and such like, and we have to be on top of that with GDPR but that’s the way the world is moving and we need to keep up.”* **(GP, 31-50 years)** |
| Automatic Motivation | Reinforcement  Emotions | Familiarity and availability | *“[Why do you use electronic systems?]... and because that’s, I suppose it’s what we’ve always used, we’ve never been told there’s anything else that can be used.”* **(Nurse, 31-50 years)**  *“I mean, sometimes when you first learn them, it is a bit daunting. You think, ‘oh’, and you’re looking through them, but once you’ve done it a few times, you get a rhythm... As I said, if you go through every box, you can’t go wrong.”* **(HCA, 31-50 years)** |
|  |  | Prompt behaviour | *“I suppose they are helpful but because you’re doing it all the time, you don’t really need the things anyway. It’s like being on a roller coaster; you know what you’re going to be asking but the prompts are good because, obviously, it’s making sure that you’ve not missed anything out. When the patient finishes and you close down the template some of the prompts are still there you think, Oh gosh, I’ve missed something here. So it is a good thing to have all the prompts in front of you.”* **(Nurse, 50+ years)**  *“They’re optional, yes... I choose to use them, yes... It’s easier and I feel like it’s more thorough, and when it’s a busy day especially, it’s nice to just have that as a prompt.”* **(HCA, 18-30 years)**  *“It’s useful for us because everything is there as I said. You could be sitting in a consultation. You’ve got everything there to hand for you... it’s all there for you if you use every tick box, and it’s helpful for when you do re-reviews.”* **(HCA, 31-50 years)** |
